# Supplementary material for: Controlled condensation by liquid contact-induced adaptations of molecular conformations in self-assembled monolayers
Source: Nat Commun. 2024 Apr 11;15:3132. doi: 10.1038/s41467-024-47507-x (PMC11009314; doi:10.1038/s41467-024-47507-x)
Supplement: Supplementary file 1 — Supplementary Information [file 41467_2024_47507_MOESM1_ESM.pdf]

Supplementary Information for

Controlled condensation by liquid contact-induced adaptations of molecular conformations in self-assembled monolayers

Guoying Bai[1*], Haiyan Zhang[1], Dong Gao[2], Houguo Fei[3], Cunlan Guo[3] Mingxia Ren[1] & Yufeng Liu[1]

[1]Tianjin Key Laboratory of Materials Laminating Fabrication and Interface Control Technology, School of Materials Science and Engineering, Hebei University of Technology, Tianjin 300401, P. R. China

[2]Key Laboratory of Hebei Province for Molecular Biophysics, Institute of Biophysics, School of Health Science & Biomedical Engineering, Hebei University of Technology, Tianjin 300401, P. R. China

[3]College of Chemistry and Molecular Sciences, Wuhan University, Wuhan, Hubei 430072, P. R. China

[*]Correspondence and requests for materials should be addressed to G. Bai (baiguoying@iccas.ac.cn).

This PDF file includes:

Supplementary Fig. 1-25

Supplementary Table 1

Supplementary Figures

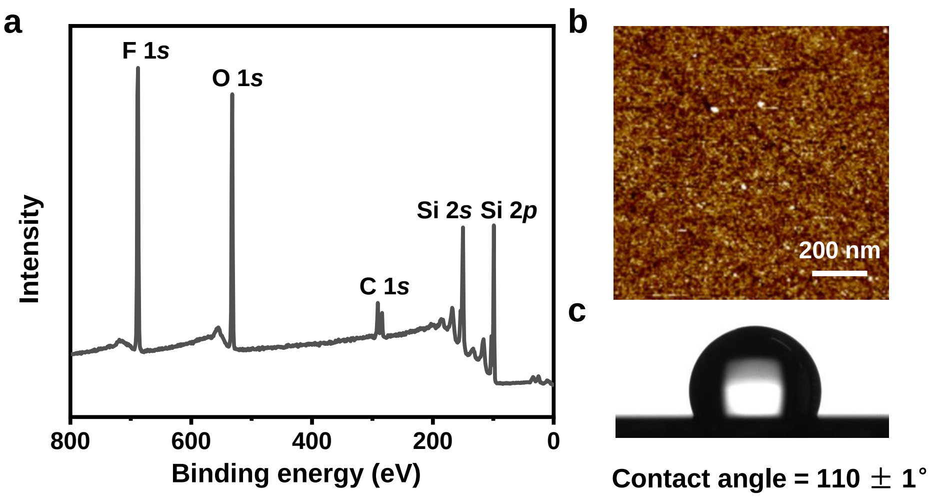

Supplementary Fig. 1. | Characterizations of Si-FDTS. a, XPS spectrum, **b**, AFM image and **c**, water contact angle of the Si-FDTS. The results suggest that the FDTS has been successfully grafted on the surface of silicon wafer.

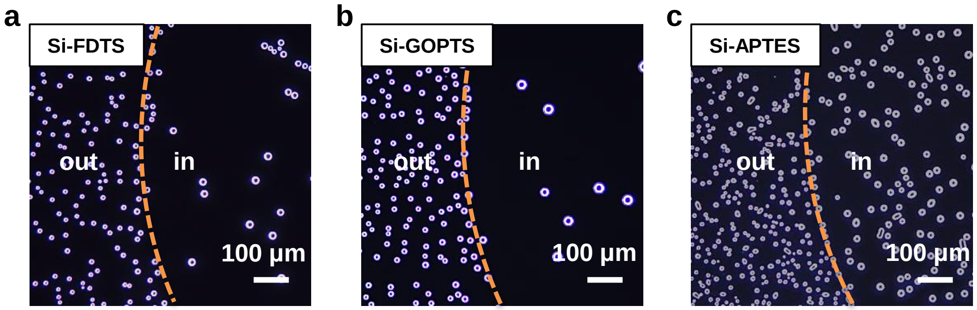

Supplementary Fig. 2 | Condensation differences between area "in" and "out" for various SAMs. Typical optical microscopic images (dark-field) of the condensed water droplets on areas "in" and "out" of **a**, Si-FDTS, **b**, Si-GOPTS and **c**, Si-APTES surfaces in the equilibrium stage of condensation (*i.e.*, both the density and diameter of the condensed water droplet reach the maximum). All the following condensation images are captured in the equilibrium stage of condensation unless particularly stated.

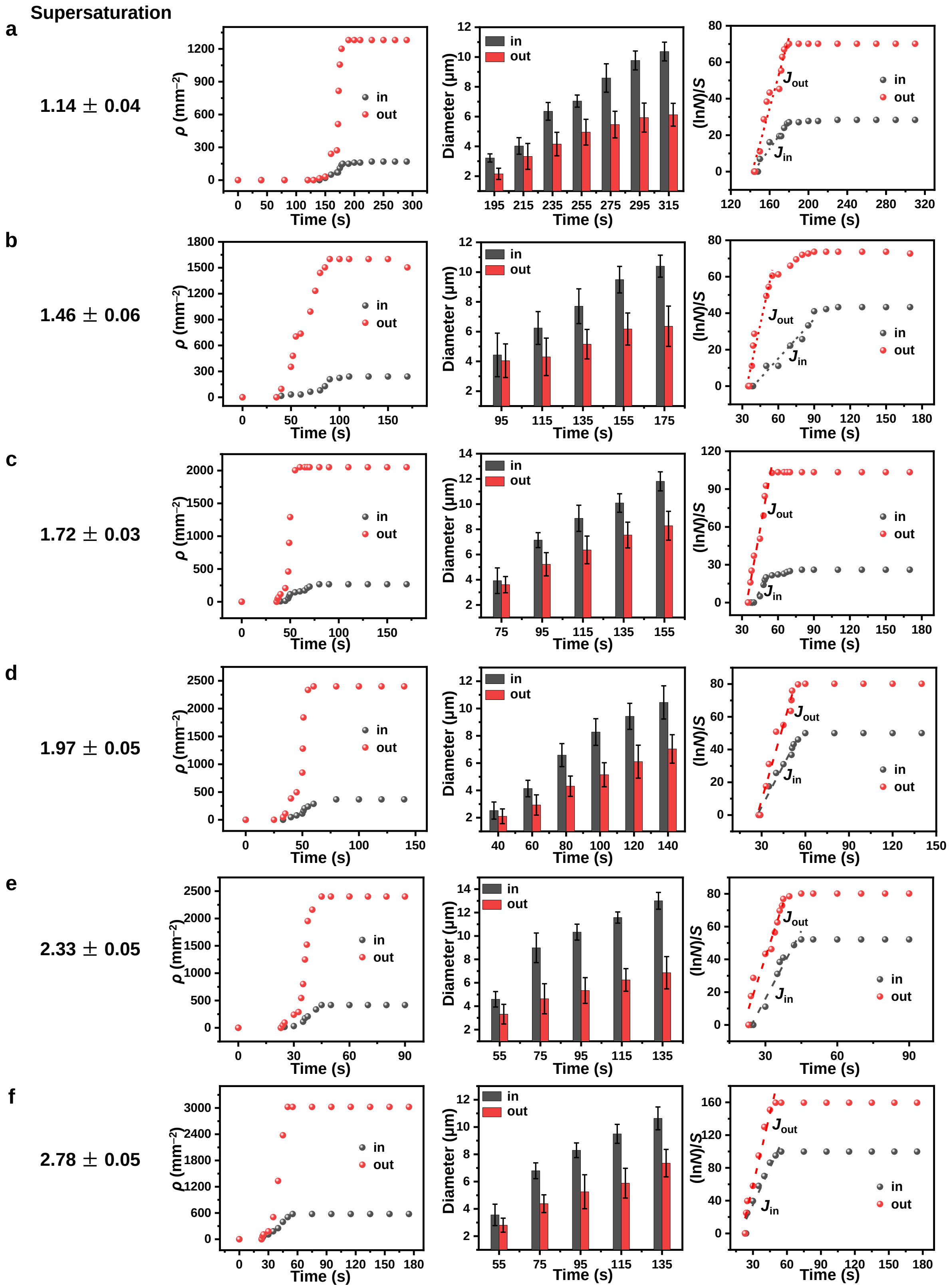

Supplementary Fig. 3 | Typical variation of ρ, condensed droplet diameter, and (ln N)/S on area "in" and "out" with time under various investigated supersaturation values ranging from

1.0 to 3.0. Time 0 represents the moment when humid N_2 starts to be injected into the sample chamber. The flow rate of the humid N_2 is fixed at 0.439±0.018 L min^{-1}. The error bars are standard deviation based on 3 measurements.

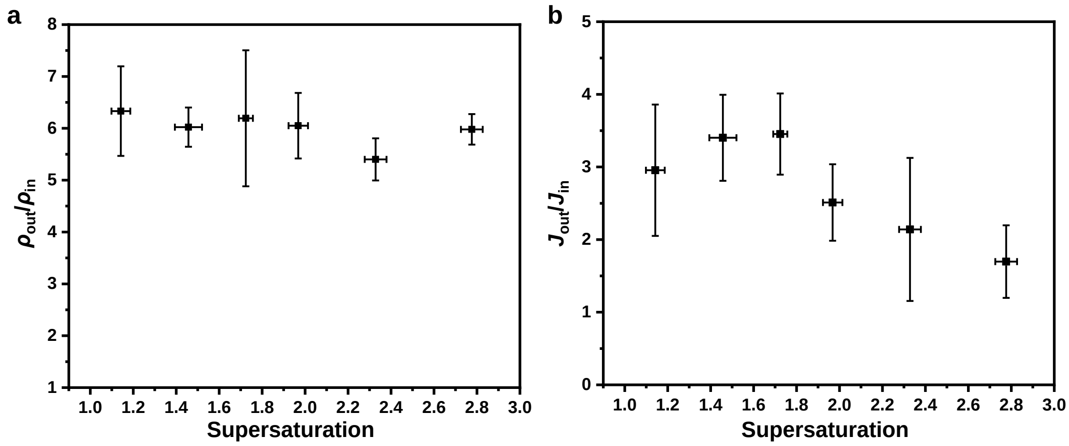

Supplementary Fig. 4 | ρ_{out}/ρ_{in} and J_{out}/J_{in} under various investigated supersaturation values. The ρ here represents the droplet density in the equilibrium stage of condensation. The error bars are standard deviation based on 3 measurements. Obviously, both the values of ρ_{out}/ρ_{in} and J_{out}/J_{in} are higher than 1, indicating the LCICD effect.

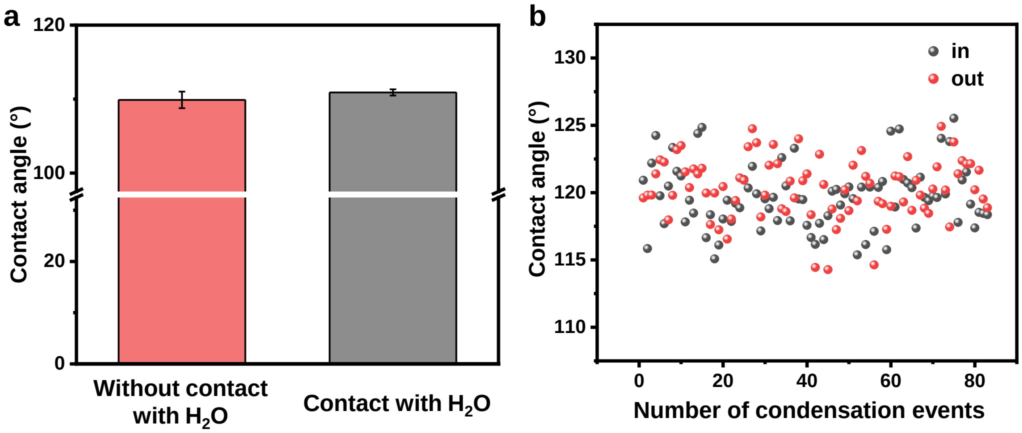

Supplementary Fig. 5 | Water contact angles on Si-FDTS surfaces that have been in contact with and without water. a, Contact angles of macroscopic water droplets (2 μL) on Si-FDTS surfaces that have been in contact with and without water. The error bar is the standard deviation based on 5 measurements. **b**, Contact angles of condensed microdroplets on the area "in" and "out".

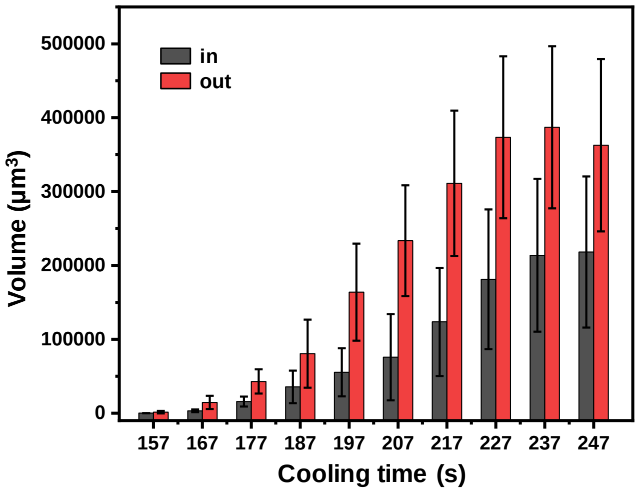

Supplementary Fig. 6 | Variation of total water droplet volume on each square millimeter surface for area "in" and "out". The volume of each water droplet (V_d) is calculated by the equation: $V_d = \pi/3(3R\text{-}H)H^2$, where R and H are the radius and height of the water droplet. H is obtained based on the water contact angle and R. The error bars of the calculated parameters are calculated according to the error propagation formulae and represent standard deviation based on 3 measurements. It is obvious that the total volume of condensed water droplet on area "in" increases slower compared with that on area "out", suggesting the dominant role of the ρ difference.

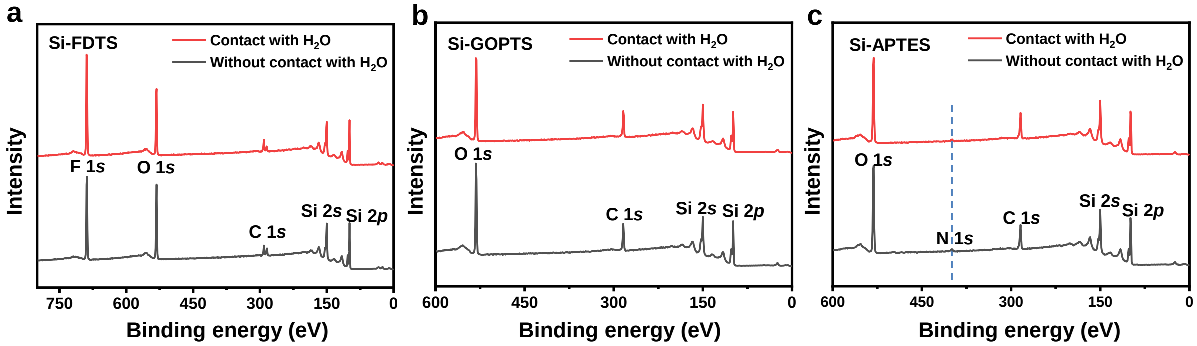

Supplementary Fig. 7 | XPS spectra of various SAMs that have been contacted with/without water. a, Si-FDTS. **b**, Si-GOPTS. **c**, Si-APTES.

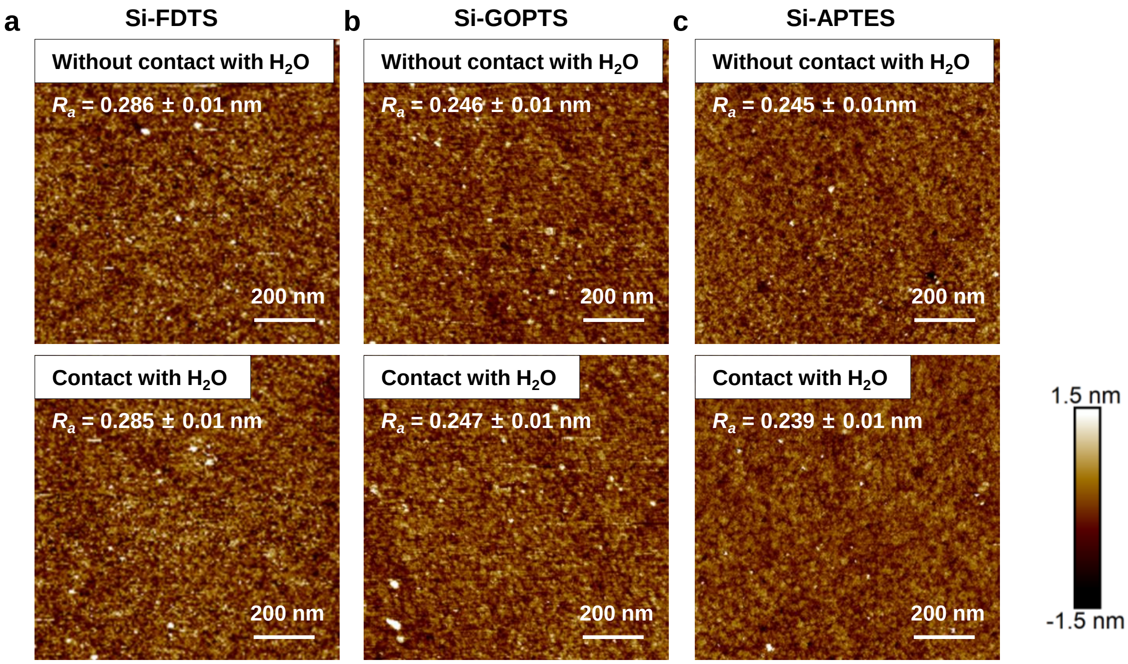

Supplementary Fig. 8 | AFM images of various SAMs that have been contacted with/without water. a, Si-FDTS. **b**, Si-GOPTS. **c**, Si-APTES. R_a means the arithmetic mean roughness.

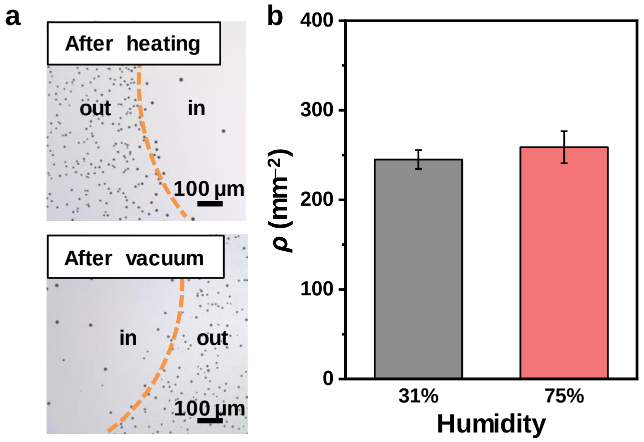

Supplementary Fig. 9 | Water condensation behaviors on Si-FDTS under various pretreatments. a, Typical optical microscopic images (bright-field) of the condensed water droplets on areas “in” and “out” of Si-FDTS surfaces which have been pretreated with water contact, and then following with heating (100 °C for 12 h) or vacuum treatment (< 10^{-3} torr for 12 h). The results show that the condensation differences between area “in” and “out” are still obvious after the heating or vacuum treatment (the treatment of the surface can remove, if any, the adsorbed water). **b**, Density of condensed water droplets on Si-FDTS which have been stored in humidistat with relative humidity of 37% or 75% for 5 h. The error bars are the standard deviation based on 3 measurements. The aim of storage under different relative humidity is to supply different degrees of water adsorption, if there is indeed water adsorption on Si-FDTS surfaces. The condensation densities on surfaces having been stored under different relative humidity show no difference.

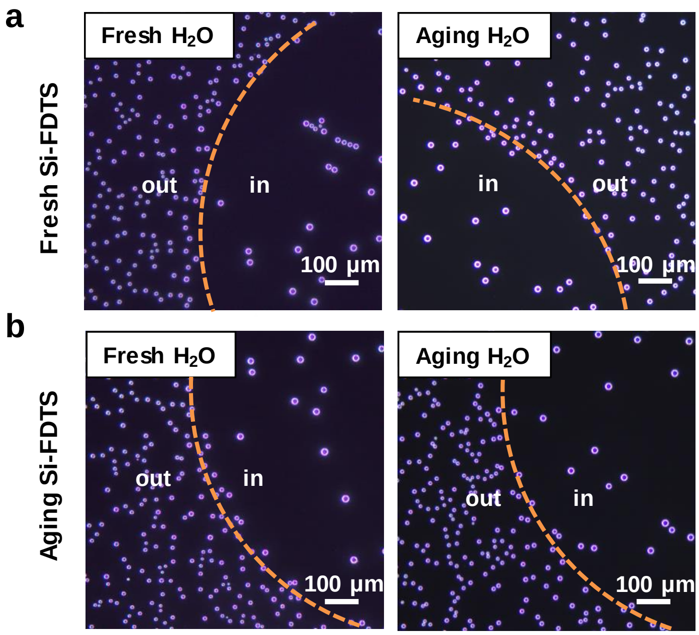

Supplementary Fig. 10 | Condensation differences between area "in" and "out" under the induction of fresh/aging water contact. Typical optical microscopic images (dark-field) of the condensed water droplets on areas "in" and "out" of fresh prepared (**a**) and aging (**b**) Si-FDTS surfaces after the induction of fresh/aging water contation. The aging Si-FDTS sample and water were obtained by storing the fresh prepared Si-FDTS and water in atmospheric environment for 4 days, respectively.

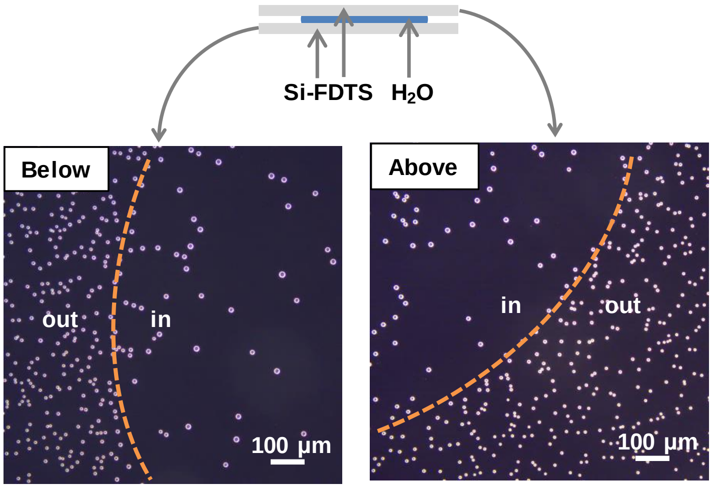

Supplementary Fig. 11 | Condensation differences between area "in" and "out" under the induction of a thin layer of water. Typical optical microscopic images (dark-field) of the condensed water droplets on areas "in" and "out" of Si-FDTS surfaces after the induction of a thin layer of water sandwiched between the two Si-FDTS surfaces.

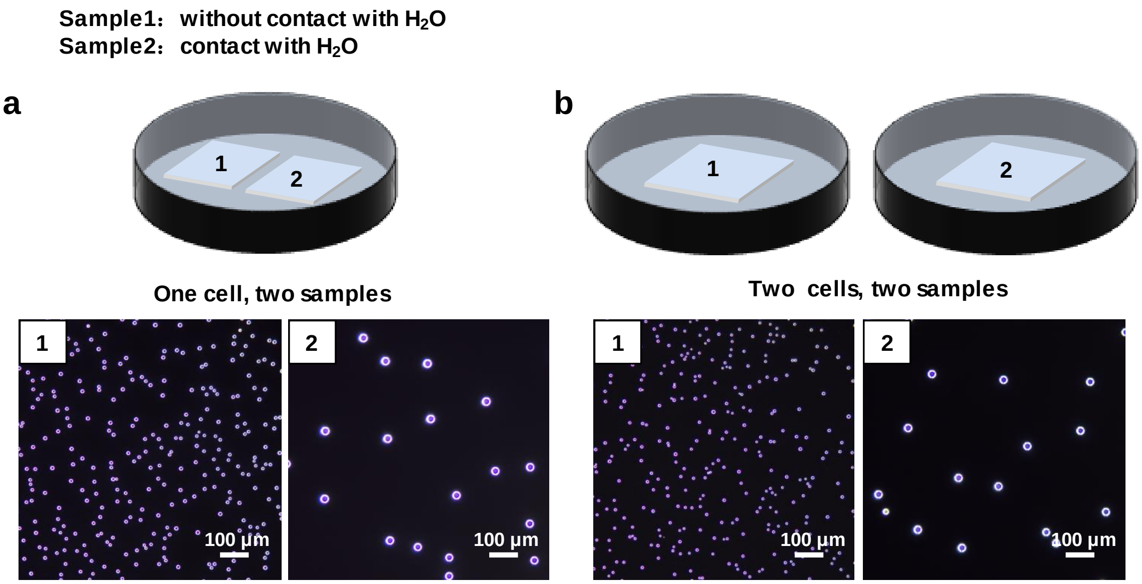

Supplementary Fig. 12 | Condensation differences observed in one cell or separate cells. a, Typical optical microscopic images (dark-field) of the condensed water droplets on Si-FDTS surfaces with/without water contation induction in the same cell (**a**) or separate two cells (**b**).

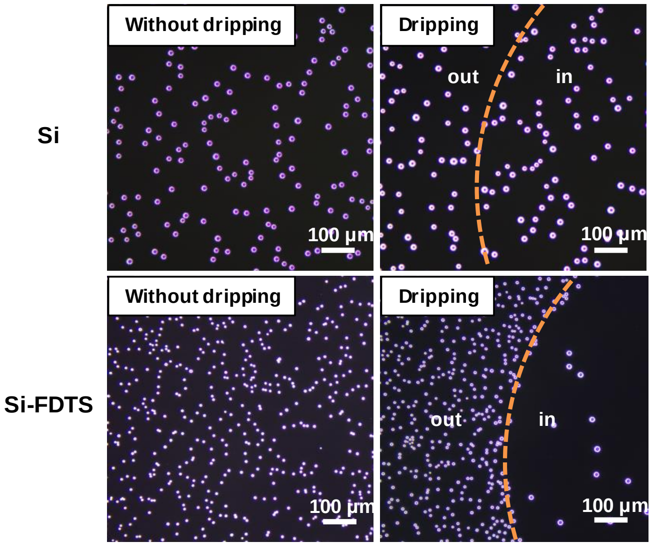

Supplementary Fig. 13 | Condensation differences between area "in" and "out" for unmodified silicon surface and Si-FDTS. Typical optical microscopic images (dark-field) of the condensed water droplets on unmodified silicon surface and Si-FDTS surfaces before and after dripping water droplet for contation induction.

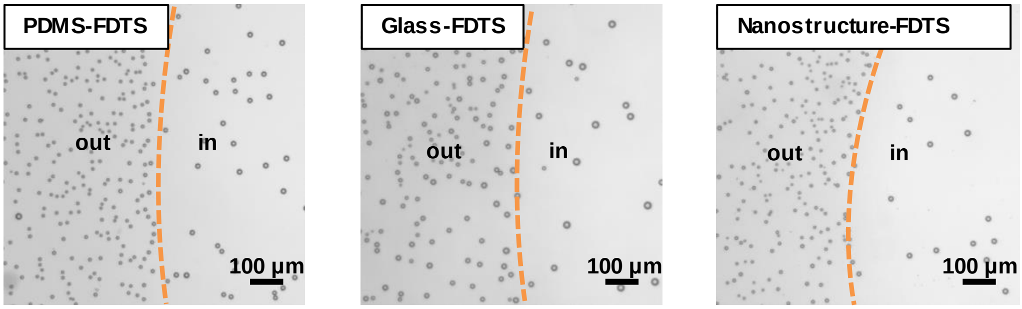

Supplementary Fig. 14 | Condensation differences between area "in" and "out" for FDTS SAM on various substrates. Typical optical microscopic images (bright-field) of the condensed water droplets on area "in" and "out" of FDTS SAM on soft PDMS/flat glass (coverslip)/nanostructured (coverslip anchored with SiO_2 nanoparticles) substrate.

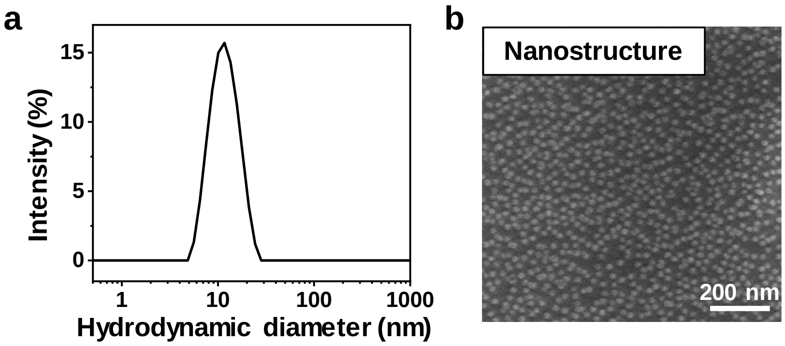

Supplementary Fig. 15 | Characterization of nanostructured surface. a, Hydrodynamic diameter distribution of SiO_2 nanoparticles. **b**, SEM image of coverslips anchored with SiO_2 nanoparticles.

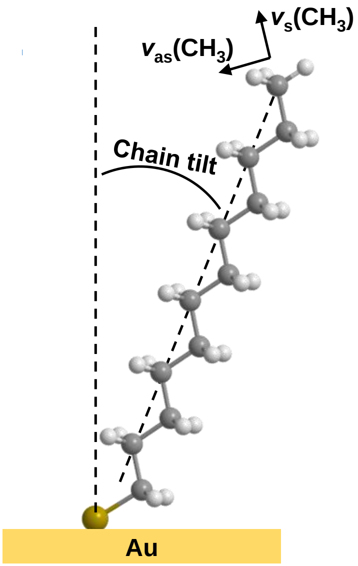

Supplementary Fig. 16 | Schematic illustration of 1-dodecanethiol adsorbing on Au surface with a hydrocarbon chain tilt of about 27 °. The arrows indicate the transition dipole moments of

the methyl symmetric/asymmetric stretching vibration modes. The intensity of each of the vibrational modes depends on the projection of its transition dipole moment along the surface normal.

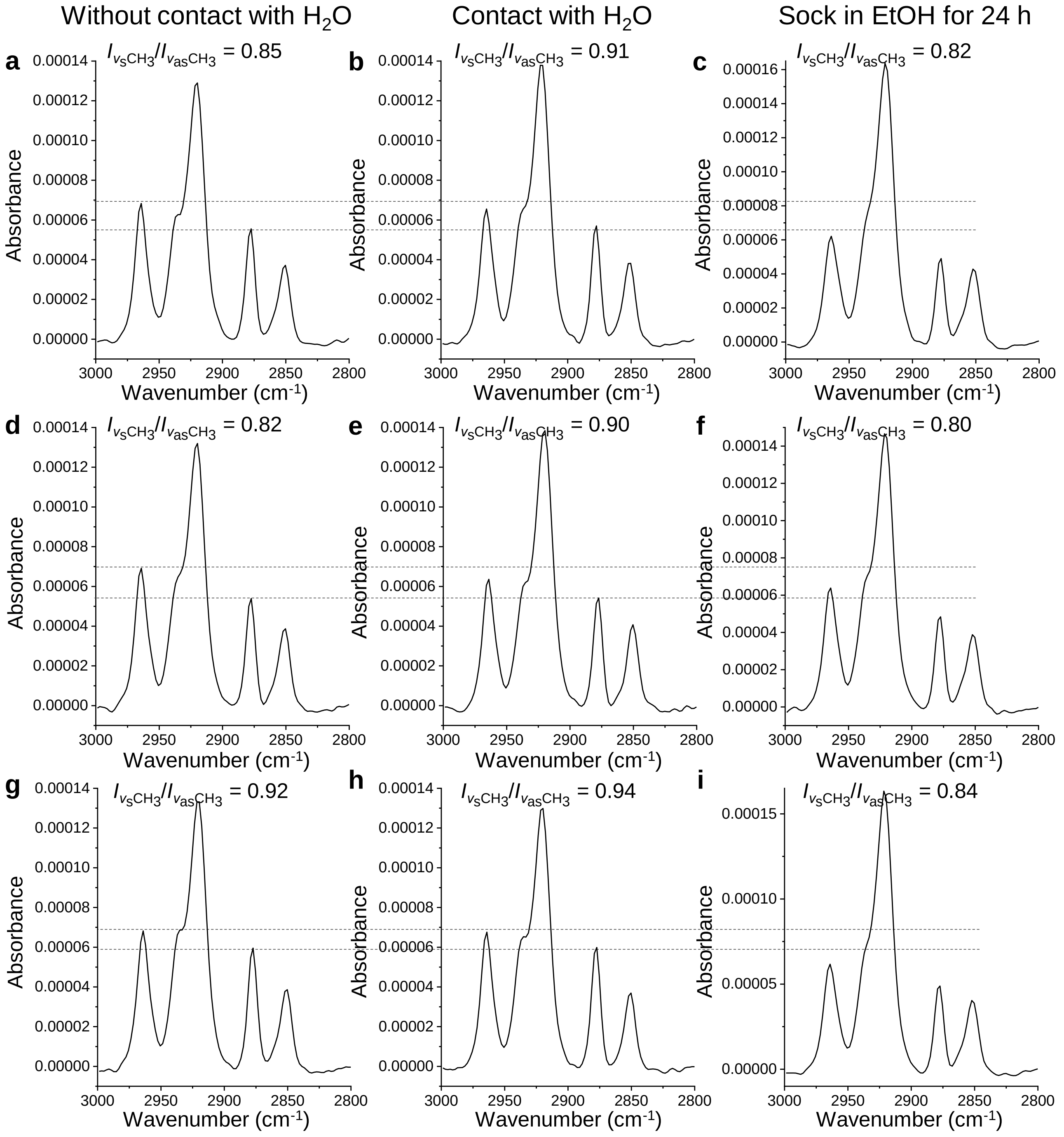

Supplementary Fig. 17 | PMIRRAS spectra of Au-C_{12} that have been sequentially pretreated without water contact (a, d, g), with water contact (b, e, h), and with EtOH soaking (c, f, i). The ratios of $I\nu_s(CH_3)/I\nu_{as}(CH_3)$ are given in the figure. The dotted lines are plotted for guidance.

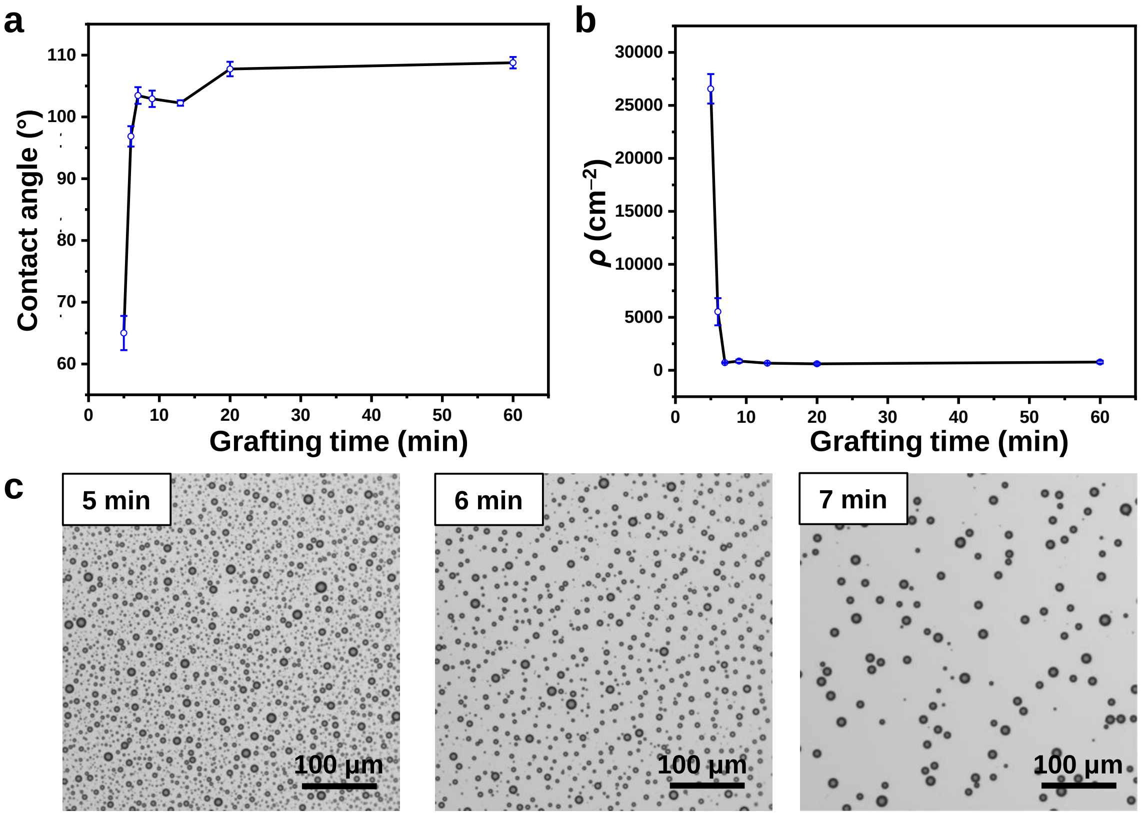

Supplementary Fig. 18 | Densities of condensed water droplets on surfaces with FDTS of various grafting densities. a, Contact angle of water on surfaces with FDTS obtained under various grafting time. The increasing water contact angle with the increasing grafting time reflects the increase of the grafted FDTS molecular density, i.e., the decrease of the FDTS defects. **b**, Variation of condensed water droplet density on Si-FDTS with grafting time. **c**, Typical optical microscopic images (bright-field) of the condensed water droplets on Si-FDTS surfaces prepared under various grafting time. All the error bars are the standard deviation based on 3 measurements.

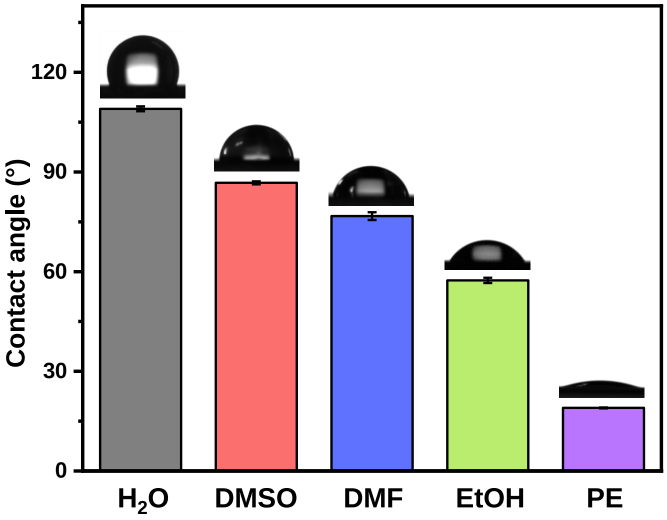

Supplementary Fig. 19 | Solvophilicity of Si-FDTS. Contact angle of various liquids of decreasing polarities on Si-FDTS. The error bars are the standard deviation based on 3 measurements.

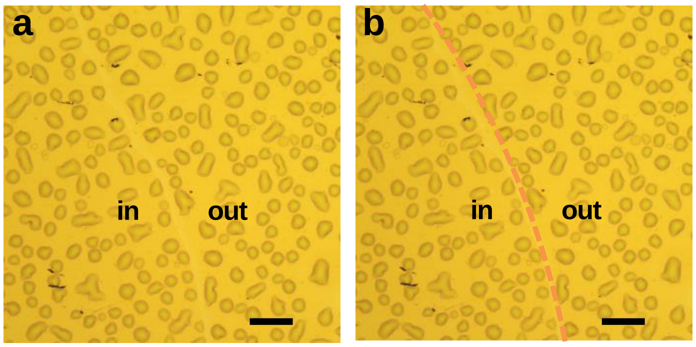

Supplementary Fig. 20 | Typical optical microscopic images (bright-field) of the condensed water droplets on area "in" and "out" of O_2 plasma-activated Au surface. The gap without condensation at the "in"-"out" area boundary is marked by a dashed curve, as shown in figure b. All the scale bars are 100 μm.

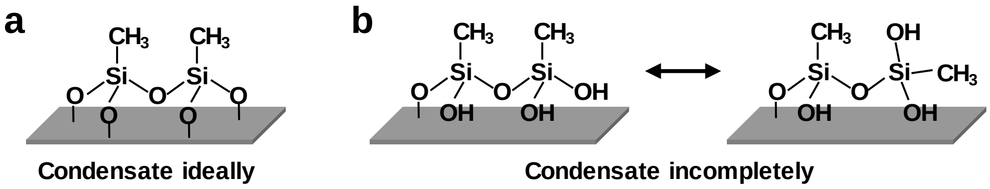

Supplementary Fig. 21 | Schematic diagram of silane's grafting states on substrate. Schematic diagram showing the Si-O condensation ideally and incompletely on substrate.

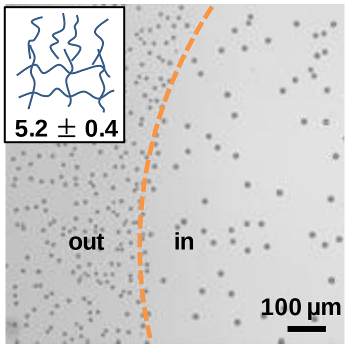

Supplementary Fig. 22 | Condensation differences between area "in" and "out" for crosslinked PDMS networks having not been washed to remove the unreacted PDMS oligomers. Typical optical microscopic images (bright-field) of the condensed water droplets on crosslinked PDMS networks having not been washed to remove the unreacted PDMS oligomers. The inset shows the structural schematic diagram of the crosslinked PDMS containing free PDMS oligomers and the value of ρ_{out}/ρ_{in}.

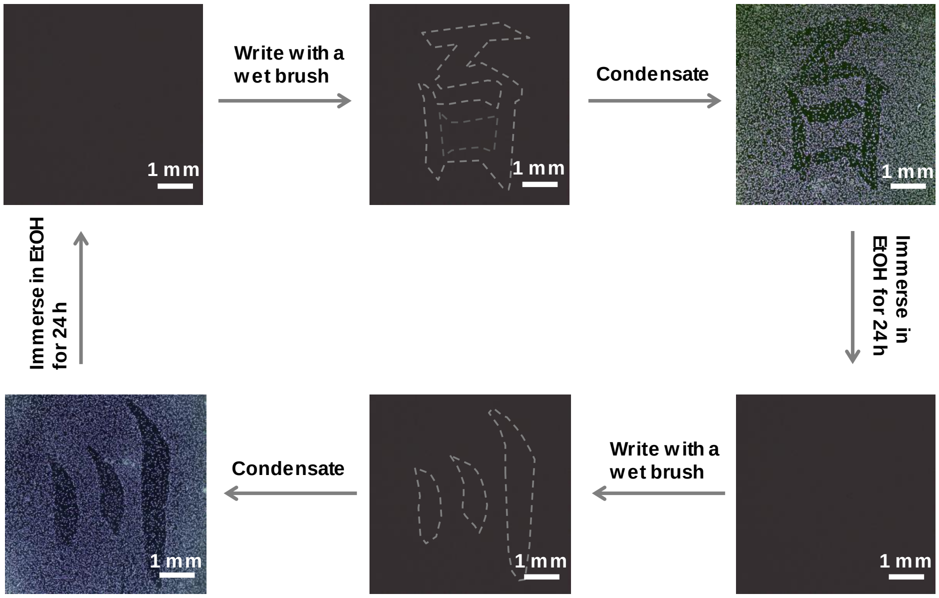

Supplementary Fig. 23 | Recyclability of the SAM surfaces (Si-FDTS here) for information storage. Typical optical microscopic images (dark-field) showing the erasability and rewritability of the stored information.

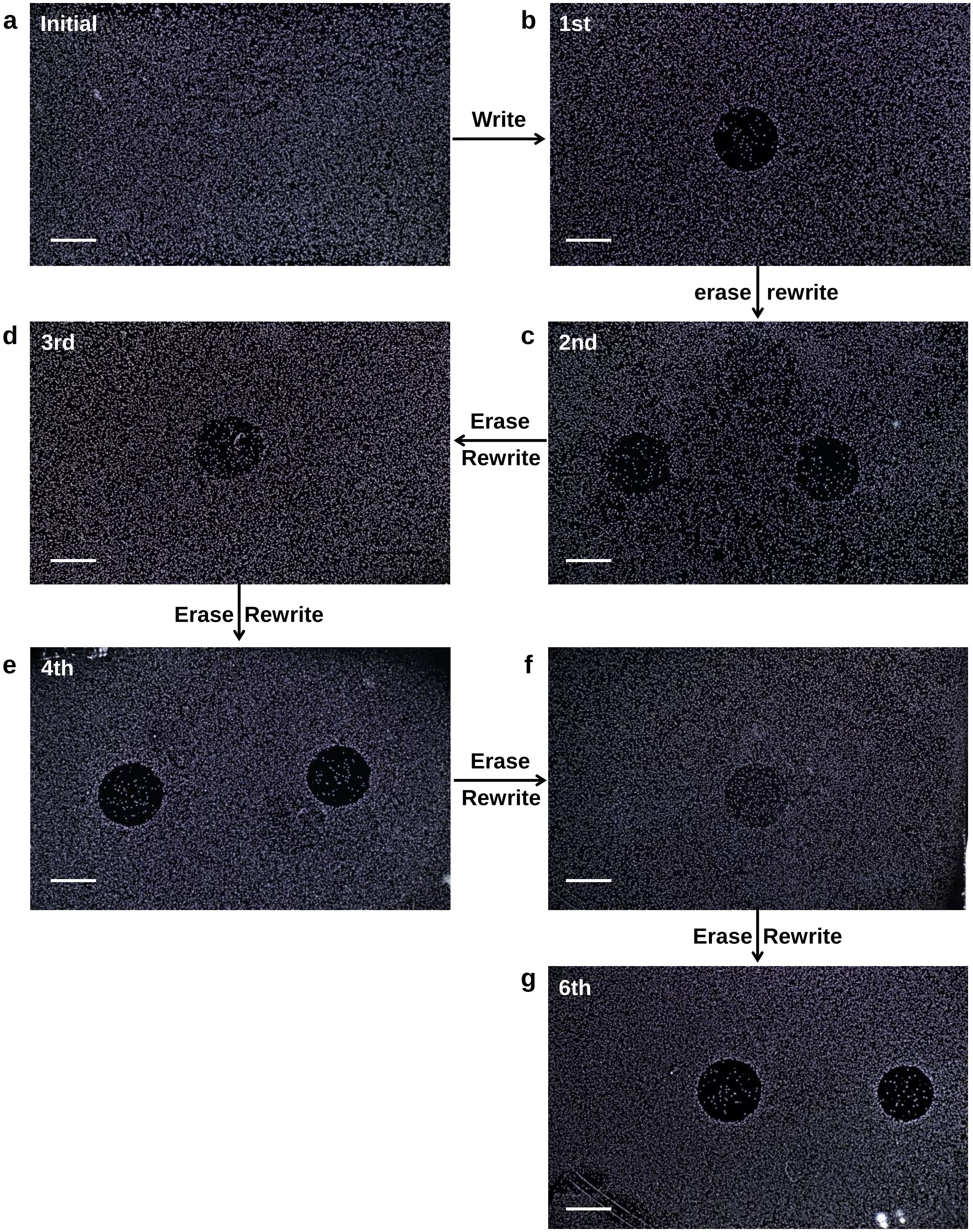

Supplementary Fig. 24 | Demonstration of multiple writing and erasing-cycles. Here alternation of single and double droplet information storage is adopted for convenience. Scale bar: 1mm.

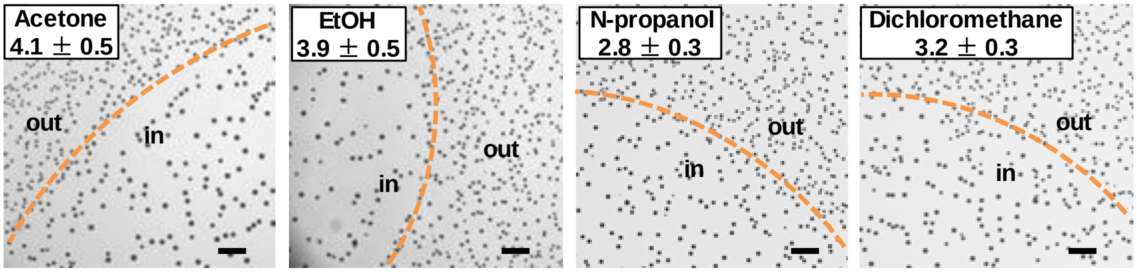

Supplementary Fig. 25 | Applicability of LCICD effect for other condensation processes beyond water. Typical optical microscopic images (bright-field) showing the condensation differences of acetone, EtOH, n-propanol and dichloromethane between area "in" and "out" on Si-FDTS. The insets show the values of ρ_{out}/ρ_{in}. All the scale bars are 100 μm.

Supplementary Table

Supplementary Table 1. Contact angle of water on a series of surfaces with segments of varying degrees of flexibility.

Surface	Si	Si-OH	Si-C_1	Si-C_2	Au	O_2 plasma -treated Au	Au-SH	Au-C_1	Au-C_{12}	Crosslinked PDMS	Coverslip	PDMS brush
Contact angle of water (mean ± s.d.) (°)	53.3 ± 0.8	12.6 ± 1.0	71.2 ± 2.9	82.6 ± 2.7	76.7 ± 1.8	< 5	77.9 ± 3.0	90.7 ± 2.6	102.4 ± 1.5	113.0 ± 1.3	9.8 ± 2.0	104.7 ± 0.3
