# Supplementary material for: Controlled condensation by liquid contact-induced adaptations of molecular conformations in self-assembled monolayers
Source: Nat Commun. 2024 Apr 11;15:3132. doi: 10.1038/s41467-024-47507-x (PMC11009314; doi:10.1038/s41467-024-47507-x)
Supplement: Supplementary file 3 — Description of Additional Supplementary Files [file 41467_2024_47507_MOESM3_ESM.pdf]

Description of Additional Supplementary Files

File Name: Supplementary Movie 1
Description: Condensation process of water on the area “in” and “out” of Si-FDTS surface.

File Name: Supplementary Movie 2
Description: Condensation processes of water on hydrophilic (Si-OH) and hydrophobic (Si-FDTS) surfaces.
